# Mitochondria–sarcoplasmic reticulum crosstalk as a modulator of skeletal muscle mass

**DOI:** 10.1007/s13105-026-01198-8

**Published:** 2026-06-30

**Authors:** Rafael A. Casuso

**Affiliations:** Department of Biosciences, Universidad Loyola Andalucía, Córdoba, Spain

**Keywords:** Atrophy, Organelle crosstalk, Calcium, Unfolded protein response, Sarcopenia, Endoplasmic reticulum

## Abstract

Preservation of skeletal muscle mass and function is a key feature of healthy ageing and relies on the tight coordination between protein synthesis and breakdown to maintain proteostatic balance. These processes impose a substantial energetic demand, highlighting the importance of mitochondrial function in skeletal muscle homeostasis. Increasing evidence indicates that mitochondria and the sarcoplasmic reticulum are functionally interconnected. Effective crosstalk between these organelles contributes to the integration of bioenergetic supply, Ca²⁺ handling, and proteostasis. Disruption of this communication network may impair adaptive stress responses, compromise protein quality control, and favour the development of anabolic resistance during ageing. This review synthesizes current evidence on mitochondria–sarcoplasmic reticulum communication. It further discusses how disruption of this crosstalk may promote anabolic resistance and skeletal muscle atrophy, with particular emphasis on its implications for age-related muscle decline.

## Key points

SR-mitochondria crosstalk emerges as a regulator of muscle mass.

Age-related proteostasis loss contributes to skeletal muscle decline.

Calcium transfer integrates energy production with protein homeostasis.

SR stress and mitochondrial dysfunction form a feed-forward loop.

## Introduction

Skeletal muscle (SKM) is the largest organ in the human body, accounting for approximately 40% of total body mass in healthy young individuals. However, SKM mass declines by ~ 1–2% per year after the fourth decade of life [1], a process that may ultimately lead to sarcopenia. Sarcopenia is defined as a progressive and generalized loss of muscle mass and strength that contributes to functional decline and disability [2]. Although low muscle strength is currently the primary diagnostic criterion, reduced muscle mass remains a central feature of the disease. Importantly, the age-related loss of SKM has consequences beyond impaired locomotion and force production. SKM also exerts key non-canonical functions, including endocrine signaling, regulation of energy expenditure, and maintenance of glucose homeostasis [3–5].

In addition, SKM constitutes the major protein reservoir in the body, and myofiber size is largely determined by the balance between protein synthesis and breakdown [6, 7]. Given that the myofiber cytoplasm is predominantly occupied by contractile proteins, even subtle alterations in protein quality control may have profound consequences for muscle structure and function. Importantly, age-related muscle dysfunction can occur despite preserved rates of protein synthesis. This is because the accumulation of damaged or misfolded proteins compromises contractile efficiency and cellular integrity [8, 9]. In this context, SKM mass and function critically depend on proteostasis. Proteostasis is a dynamic process through which protein function is maintained via the coordinated regulation of protein synthesis, folding, trafficking, and degradation [10]. The progressive loss of proteostatic control with ageing contributes to impaired proteome maintenance and increased proteotoxic stress. This ultimately promotes muscle atrophy and sarcopenia [11].

The sarcoplasmic reticulum (SR) is the key organelle regulating protein folding and calcium homeostasis. Therefore, it is likely to play a central role in the regulation of SKM mass during ageing. In parallel, age-related mitochondrial dysfunction has been closely linked to impaired proteostasis [11]. Several reviews have addressed the role of mitochondria in the regulation of SKM mass and function [8, 12]. However, the molecular crosstalk between mitochondria and the SR remains poorly defined. This review discusses the molecular interactions between the SR and mitochondria. It also examines how their functional integration may critically influence SKM mass and function across ageing.

## Protein synthesis regulation: an overview

The mechanistic target of rapamycin (mTOR) is a serine/threonine kinase that integrates nutrient availability, growth factor signalling, and cellular stress to regulate anabolic processes. As the central regulator of protein synthesis, mTOR signalling has been extensively reviewed elsewhere, and the reader is referred to these studies for a comprehensive description [13–16]. Briefly, mTOR complex 1 (mTORC1) promotes protein synthesis by activating signalling pathways that control mRNA translation and ribosome biogenesis.

Regulation of translation initiation is primarily mediated through phosphorylation of eukaryotic initiation factor 4E (eIF4E)-binding proteins (4E-BPs), which relieves their inhibitory interaction with eIF4E and allows assembly of the translation initiation complex [14]. In parallel, mTORC1 activates p70 S6 kinase 1 (S6K), triggering a phosphorylation cascade that further enhances mRNA translation [17]. Consistent with this, mTORC1-dependent phosphorylation of S6K and inhibition of 4E-BP1 represent core mechanisms by which mTOR signalling increases protein synthesis [15].

One of the best-characterized upstream regulators of mTORC1 is the tuberous sclerosis complex (TSC). TSC acts as a molecular brake on the Rheb–mTORC1 axis under conditions of nutrient or energy stress. In skeletal muscle, anabolic stimuli such as insulin signalling activate AKT. This leads to inhibition of TSC2 and subsequent activation of mTORC1, thereby promoting muscle hypertrophy [16, 18]. Conversely, AMP-activated protein kinase (AMPK) phosphorylates TSC2 in response to energetic stress. This results in suppression of mTORC1 activity [19].

Notably, protein synthesis is among the most ATP-demanding cellular processes and is highly sensitive to reductions in cellular energy availability [20]. Accordingly, sustained protein synthesis requires tight coordination between anabolic signalling and cellular energy production. Therefore, impaired energy provision may contribute to the anabolic resistance observed in aged muscle. Anabolic resistance is defined as a blunted stimulation of muscle protein synthesis in response to anabolic stimuli, such as amino acid availability [21].

## Mitochondria: beyond ATP production

Mitochondria are eukaryotic organelles composed of an outer mitochondrial membrane (OMM), an inner mitochondrial membrane (IMM), and a soluble matrix. The OMM mediates metabolic exchange and inter-organelle interactions. In contrast, the IMM is organized into boundary membranes and cristae that harbour the electron transport chain (ETC) and support oxidative phosphorylation (OXPHOS) [22, 23].

It has long been established that mitochondria constitute the primary site of aerobic ATP synthesis in eukaryotic cells [24]. However, advances over the past decades have substantially expanded this classical view. These advances have revealed mitochondria as multifunctional organelles involved in biosynthetic pathways [25]. They also act as central signalling hubs capable of influencing cellular fate decisions [26]. In addition, increasing evidence supports extensive mitochondrial crosstalk with other cellular compartments, including the endoplasmic reticulum and the nucleus. This crosstalk enables coordinated regulation of cellular homeostasis [27–29].

In this context, mitochondria have been proposed to function as cellular transducers. They convert diverse signals, such as ionic fluxes, protein interactions, and changes in cellular energy state, into coordinated genetic and metabolic responses [30]. This transducing capacity relies on both inter-mitochondrial communication and dynamic interactions with other organelles. It also aligns with the emerging role of mitochondria as central regulators of systemic health and ageing [31].

## ER stress and the unfolding protein response regulates protein synthesis

The unfolded protein response (UPR) is activated in response to the accumulation of unfolded or misfolded proteins within the endoplasmic reticulum (ER) [32]. Throughout this article, “ER” refers to broadly described mechanisms of ER stress and UPR signaling. “SR” is used when these mechanisms are specifically discussed in SKM.

The UPR is mediated by three ER transmembrane sensors [32]: RNA-dependent protein kinase-like ER eukaryotic translation initiation factor 2α kinase (PERK), inositol-requiring enzyme 1 (IRE1), and activating transcription factor 6 (ATF6). Under basal conditions, these sensors are maintained in an inactive state through their association with the ER chaperone BiP/glucose-regulated protein 78 (GRP78).

Upon SR stress, activation of the UPR can lead to adaptive responses aimed at restoring proteostasis. These responses preserve muscle mass, metabolic homeostasis, and Ca²⁺ handling. However, if SR stress is unresolved or chronically sustained, UPR activation can lead to maladaptive signalling. This promotes SKM atrophy, activation of proteolytic pathways, and inhibition of protein synthesis [33, 34].

In its adaptive phase, UPR activation transiently reduces the burden of newly synthesized proteins entering the ER. It also increases folding capacity, ER-associated quality control, and stress-resolution mechanisms [35]. This is particularly evident for PERK, whose phosphorylation of eIF2α (eukaryotic initiation factor-2α) acutely attenuates global translation. At the same time, it allows selective ATF4 (activating transcription factor 4)-dependent expression of stress-responsive genes, thereby coupling protein synthesis rates to ER folding capacity [35]. Such transient UPR activation should therefore be distinguished from chronic or unresolved SR stress. This is because low or basal levels of SR stress may promote hormetic adaptations involving autophagy, mitochondrial function, and antioxidant defences [33, 34, 36]. Moreover, ATF6 appears to contribute predominantly to the adaptive arm of the UPR in SKM. It does so by increasing the expression of chaperones and protein quality-control genes, thereby enhancing folding capacity and facilitating recovery from SR stress. Indeed, in myofibres, ATF6 has also been linked to contraction-induced adaptation through functional interaction with PGC-1α, a master regulator of mitochondrial biogenesis [36].

Conversely, sustained PERK–eIF2α–ATF4 signalling can become maladaptive when ATF4/CHOP (C/EBP homologous protein)-dependent programmes persist, leading to prolonged translational repression, activation of proteolytic systems, and muscle atrophy [37]. Consistent with this dual role, PERK activity is required for the maintenance of SKM mass and function under basal conditions, whereas sustained ATF4 activity has been implicated in age-related reductions in skeletal muscle protein synthesis, strength, quality, and mass [37, 38]. Importantly, IRE1 may also contribute to this adaptive-to-maladaptive transition: whereas IRE1–XBP1 (X-box- binding protein-1) signaling supports SR protein folding, SR-associated degradation, and proteostatic recovery; prolonged IRE1 activation can engage inflammatory signalling, thereby linking unresolved SR stress to muscle dysfunction [33, 34, 36].

## The role of calcium in mitochondria-sarcoplasmic reticulum crosstalk

Beyond its function in Ca²⁺ storage and release, the SR plays a critical role in protein turnover as the primary organelle responsible for protein folding. As discussed above, protein synthesis is among the most energy-sensitive processes in mammalian cells [20], highlighting the importance of efficient SR–mitochondria communication to match local energetic supply with proteostatic demand.

Within SKM, intermyofibrillar mitochondria lie near calcium release units (CRUs), highly organized junctional domains in which a T-tubule is flanked by two SR membranes that serve as intracellular calcium reservoirs [39]. Upon T-tubule depolarization, a voltage-gated receptor, the dihydropyridine receptor, undergoes a conformational change, thereby opening ryanodine receptor isoform 1 (RyR1) in the sarcoplasmic reticulum and leading to Ca²⁺ release. In this scenario, mitochondrial Ca²⁺ uptake mainly occurs through two pathways. The first involves direct coupling between RyR1 and VDAC (voltage-dependent anion channel), an outer mitochondrial membrane protein involved in the transport of calcium, among other molecules [40]. The second pathway involves the so-called mitochondria-associated membranes (MAMs), which are contact sites between the SR and mitochondria that allow direct Ca²⁺ efflux from the SR to mitochondria. Within SKM, MAMs have been proposed to be formed by a complex comprising IP3R (inositol 1,4,5-trisphosphate receptor), GRP75 (75-kDa glucose-regulated protein), and VDAC [41], as well as mitofusin 2 (MFN2) as a tethering protein [42]. Subsequently, Ca²⁺ import into the mitochondrial matrix occurs through the mitochondrial calcium uniporter complex (MCU), which tightly regulates Ca²⁺ uptake [43]. Under physiological stimuli, Ca²⁺ is then released from mitochondria by NCLX, the Na⁺/Ca²⁺/Li⁺ exchanger, thereby preventing excessive Ca²⁺ retention, which may otherwise lead to mitochondrial permeability transition pore opening and apoptotic signaling.

Ca^2+^ uptake enhances mitochondrial function as it is an allosteric regulator of the tricarboxylic acid (TCA) cycle. Indeed, Ca²⁺ enhances the activity of several mitochondrial matrix dehydrogenases, including isocitrate dehydrogenase [44], α-ketoglutarate dehydrogenase [45], and pyruvate dehydrogenase [46]. Thus, enhancing NADH and FADH_2_ generation which ultimately fuels the electron transport chain (ETC) for oxidative phosphorylation.

**Fig. 1:**
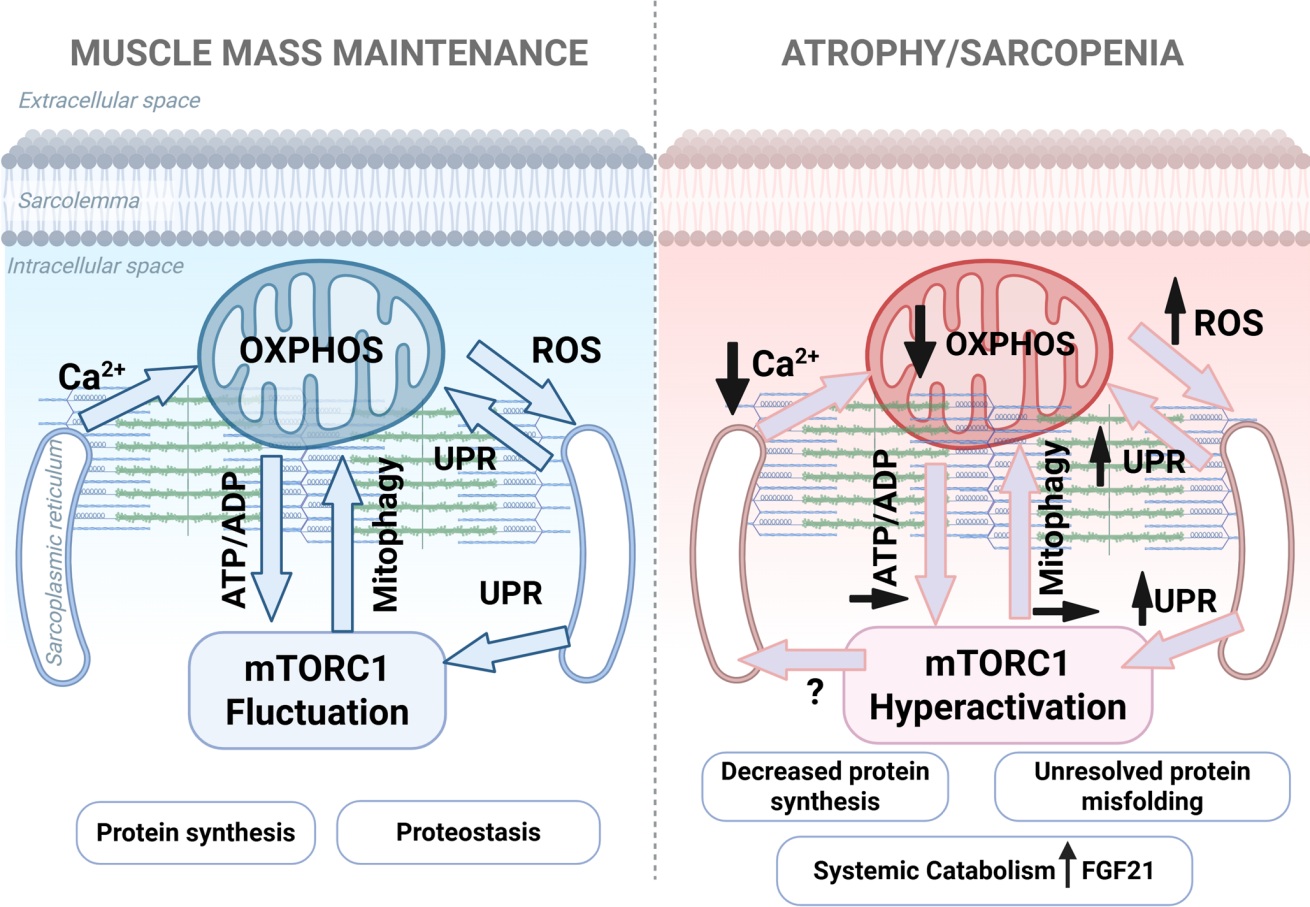
Sarcoplasmic reticulum-mitochondria crosstalk. Left, normal communication between organelles is associated with transient mTORC1 activation and muscle mass maintenance. Right, altered communication between organelles is associated with mTORC1 hyperactivation and atrophy/sarcopenia. Arrows denote increased or decreased signals. FGF21; fibroblast Growth Factor 21; OXPHOS, oxidative phosphorylation; ROS, reactive oxygen species; UPR, unfolded protein response. Created in https://BioRender.com

In this scenario, physiological Ca²⁺ transfer from the SR to mitochondria enhances local mitochondrial ATP production. This supports energy-demanding SR functions that are essential for maintaining Ca²⁺ homeostasis and proteostatic balance (Fig. 1) [47]. Consistent with this concept, structural and functional coupling between the SR and mitochondria is markedly impaired in aged SKM. In rodents, ageing is associated with a reduced number of calcium release units, SR–mitochondria uncoupling, and diminished mitochondrial Ca²⁺ uptake compared with young muscle [48]. Similar alterations have been reported in humans, where ageing is characterized by a lower frequency of calcium release units and increased spatial separation between the SR and mitochondria [49, 50].

Importantly, age-related SR–mitochondria uncoupling can be partially reversed by exercise training, an adaptation that occurs concomitantly with increased expression of optic atrophy 1 (OPA1) [51]. OPA1 is a mitochondrial inner membrane GTPase that regulates cristae architecture and inner mitochondrial membrane fusion [52]. Within aged SKM, mitochondrial Ca²⁺ uptake appears to be reduced [53]. This reduction has been linked to impaired mitochondrial function, excessive reactive oxygen species (ROS) production, oxidative damage, and loss of muscle mass [53]. This is consistent with reduced MAMs contacts reported in aged SKM in both rodents and humans [49, 50, 54]. However, it should be noted that acute disruption of the SR Ca²⁺ release machinery causes maladaptive UPR activation, leading to SKM atrophy [55]. Notably, this was accompanied by increased mitochondrial Ca²⁺ uptake through MAMs contacts and enhanced ROS production [55]. Whether this represents a homeostatic mechanism aimed at resolving UPR activation remains unknown. Nevertheless, these observations highlight that SR-to-mitochondria Ca²⁺ efflux is altered under different pathophysiological conditions associated with muscle mass loss.

Together, these findings support the existence of a bidirectional, feed-forward loop between the SR and mitochondria. In this loop, dysfunction of either organelle propagates stress signalling to the other, ultimately compromising proteostasis and SKM function (Fig. 1).

## The role of mitochondria-SR crosstalk in mTORC1 hyperactivation in aged muscle

ATF4 and mTORC1 are temporally coordinated in a dynamic, oscillatory manner, whereby ATF4 activation promotes a transient catabolic and autophagic state that suppresses mTORC1 activity. This phase is followed by a decline in ATF4 levels, allowing amino acid–dependent reactivation of mTORC1 and restoration of protein synthesis [56].

This regulatory interplay is particularly relevant in ageing, as mTORC1 hyperactivation has been consistently observed in sarcopenic SKM, and its modulation has been shown to preserve muscle mass [57]. In both murine and human muscle fibres, sustained mTORC1 activation is associated with the accumulation of structurally and functionally aberrant mitochondria [58]. This likely reflects enhanced mitochondrial biogenesis in the absence of adequate quality control mechanisms, such as mitophagy [58]. This uncontrolled mitochondrial stress can, in turn, further reinforce mTORC1 activation, establishing a maladaptive feedback loop [59].

Chronic mTORC1 activation in SKM has also been shown to induce SR stress and ATF4-dependent secretion of fibroblast growth factor 21 (FGF21), shifting muscle from a predominantly anabolic state towards a stress-adaptive endocrine phenotype [60]. In line with the emerging concept of mitochondria as signal transducers, FGF21 acts as a mitokine, conveying local mitochondrial stress to the systemic level and promoting a catabolic-like state [61].

Pan-inhibition of the UPR lead to blunted mTORC1 signaling resulting in exacerbated muscle loss in a mouse model of cancer cachexia [62]. While this may not reflect the mTROC1 hypercativation model described for aged muscle. It highlights that UPR can regulate muscle mass through mTORC1.

In this context, the adaptive arms of the UPR (i.e. PERK and ATF6 arms) seem of importance in regulating both mitochondrial function and mTORC1-induced protein synthesis. For instance, in addition to the regulatory interplay between ATF4 and mTROC1 described previously, the PERK-ATF4 arm of the UPR is known to enhance mitochondria function (see next section for details). The importance of this crosstalk in SKM has been described in a study showing that chronic SR stress disrupts SR–mitochondria communication through sustained activation of the PERK–ATF4 arm of the UPR [63]. This leads to mTORC1 hyperactivation, increased FGF21 secretion, and muscle catabolism [63]. Notably, pharmacological inhibition of mTORC1 in this model fails to restore SR–mitochondria coupling or rescue the muscle phenotype [63]. Suggesting that mTORC1 hyperactivation might be a downstream consequence of impaired organelle crosstalk rather than the primary driver of muscle degeneration (Fig. 1). Whether similar regulation occurs by the ATF6 is less evident. For instance, ATF6 has a modulatory effect in exercise-induced SKM mitochondrial biogenesis [64]. In addition, myocyte mTROC1-related hypertrophy is modulated through ATF6 signaling [65].

Here it is presented a scenario where SR stress can communicate to mitochondria through UPR signaling. Disruption of this communication could lead to muscle waste and atrophy in a mechanism that might involve mTORC1 hyperactivation.

## SR stress impact mitochondrial intrinsic function

While ER stress has been shown to modulate mitochondrial function and, consequently, cellular homeostasis, the molecular mechanisms underlying this crosstalk remain incompletely defined. Elegant work has demonstrated that ER stress induced by limited glucose availability activates the PERK–ATF4 arm of the UPR [66]. Triggering adaptive remodelling of mitochondrial cristae and reorganization of the electron transport chain (ETC) supramolecular architecture to enhance mitochondrial ATP production [66]. Mechanistically, this response is mediated by ATF4-dependent induction of the supercomplex assembly factor SCAF1 [66]. SCAF1 acts as a key molecular effector linking ER stress to an increased abundance of supercomplexes, thereby improving bioenergetic efficiency [66]. Isolated ETC complexes can assemble into supercomplexes to meet the energy requirements of the cell [67]. Increased supercomplex abundance has been described in human SKM in response to both acute [68] and chronic [69] exercise. This highlights the importance of these structures in modulating SKM metabolism.

Consistent with this concept, loss of SCAF1 results in impaired mitochondrial respiration and reduced body mass in zebrafish [70]. Notably, this phenotype can be rescued by increased nutrient availability without restoring ETC organization [71]. Indicating that ETC supramolecular architecture sets the energetic cost of growth rather than acting as an absolute determinant of ATP production. In addition, chronic disturbances in SR Ca²⁺ handling promote mitochondrial dysfunction through sustained increases in mitochondrial ROS production, ultimately contributing to muscle atrophy [55]. Although the precise molecular mechanisms remain unresolved. This phenotype may reflect maladaptive alterations in ETC supramolecular organization which regulates mitochondrial ROS production [72, 73].

Importantly, cytosolic Ca²⁺ dynamics also play a central role in physiological SKM adaptation. Acute high-intensity exercise induces transient fragmentation of the ryanodine receptor (RyR1), reducing force production due to impaired Ca²⁺ release [71]. A similar RyR1 fragmentation phenotype is observed in aged skeletal muscle and has been linked to increased mitochondrial ROS production [74, 75]. Notably, antioxidant treatment prevents RyR1 fragmentation but simultaneously blunts the induction of genes required for muscle adaptation [71]. Highlighting the dual role of ROS as both signalling molecules and mediators of dysfunction. Further supporting this framework, acute cytosolic Ca²⁺ transients during high-intensity contractions increase mitochondrial respiration in human skeletal muscle [76]. Which may be due to a reorganization of ETC towards supercomplex assembly [76].

Collectively, these observations suggest that dynamic reorganization of the ETC supramolecular structure enables mitochondria to flexibly adapt their bioenergetic output in response to SR-derived signals. Disruption of this adaptive plasticity may shift skeletal muscle toward a metabolically inefficient, anabolic-resistant state, providing a mechanistic framework that is particularly relevant to age-related muscle atrophy.

## Conclusion

The evidence reviewed here supports the concept that SKM mass and function are influenced by coordinated crosstalk between the sarcoplasmic reticulum and mitochondria. This crosstalk contributes to the integration of proteostasis, Ca²⁺ handling, and bioenergetic supply. Disruption of this organelle network with ageing may promote maladaptive stress signalling, anabolic resistance, and muscle atrophy, ultimately contributing to the development of sarcopenia. This model also raises the possibility that mTORC1 hyperactivation may reflect a downstream readout of disrupted organelle homeostasis rather than acting solely as a primary driver of the process.

However, further mechanistic studies are needed to determine whether altered SR–mitochondria communication is a causal driver, a compensatory response, or a consequence of ageing muscle dysfunction. Once these mechanisms are clarified, targeting pathways that preserve or restore SR–mitochondria integration may represent a promising strategy to mitigate age-related muscle loss. Defining these mechanisms will be essential to refine therapeutic targets and distinguish adaptive from maladaptive SR–mitochondria remodelling in ageing muscle.

## Data Availability

No datasets were generated or analysed during the current study.
